# A clinical and biochemical study to estimate the salivary alpha-amylase level in post-menopausal women with psychosomatic disorders

**DOI:** 10.4314/ahs.v25i1.41

**Published:** 2025-03

**Authors:** Kumuda Rao, Suchetha Kumari, Shishir Ram Shetty, Subhas G Babu, Renita Lorina Castelino

**Affiliations:** 1 Nitte (deemed to be University) A B Shetty Memorial Institute of Dental Sciences, Department of Oral Medicine and Radiology Mangalore- 575018; 2 Nitte (deemed to be University) K.S. Hegde Medical Academy, Department of Biochemistry, Deralakatte, Mangalore- 575018; 3 Department of Oral and Craniofacial Health Sciences, University of Sharjah

**Keywords:** Salivary Alpha-Amylase, Post-menopausal women, Psychosomatic disorder

## Abstract

**Background:**

The salivary alpha-amylase (sAA) is a key player in the oral cavity's starch digestion process. Only in the last decade has this enzyme come under greater scrutiny as a Sympathetic Nervous System (SNS) stress marker. Acute stress has been demonstrated to cause a rise in sAA, which is associated with norepinephrine levels in the blood. As an analogous marker of Sympathoadrenal Medullary System (SAM) activity reflecting the changes during acute psychosocial stress, very few studies have directly demonstrated the sensitivity of salivary alpha-amylase levels to changes in catecholamine levels in the blood.

**Methods:**

Extraoral and intraoral examination was conducted on 100 post-menopausal women to check for psychosomatic disorders/lesions. The ‘Spit Technique’ was used to collect Unstimulated saliva from individuals who had clinically identified Psychosomatic disorders or lesions. The ELISA technique was utilized to estimate salivary Alpha-Amylase.

**Results:**

The outcomes were statistically significant since they demonstrated that postmenopausal women with clinically confirmed Psychosomatic disorders/lesions had greater levels of salivary Alpha-Amylase.

**Conclusion:**

The findings of this study suggest that all post-menopausal women should undergo periodic examination for acute stress. When accompanied with the clinical presence of psychosomatic disorder/lesions, salivary estimation of alpha-amylase may be utilized as a diagnostic tool for women enduring postmenopausal psychological anguish.

## Introduction

German psychiatrist Heinroth first used the term “psychosomatic” in 1818. ‘The term “Psychosomatic Medicine” was first described by Felix Deutsch, in 1922^1^. Psyche stands for the mind, and soma is the word for the body^2^. “Disorders characterised by physiological changes that originate, at least in part, from emotional factors” is the definition of psychosomatic disorders. The mouth area is directly or symbolically connected to the primary desires and passions and is associated with potential bodily manifestations of psychological genesis^3^,^4^.

Traditionally, psychiatric diseases have been divided into two primary categories: organic and functional. As in dementia or delirium, known physical causes of organic diseases can be identified. They make up a sizable portion of psychiatric illness in functional illnesses like schizophrenia where no physical causes were present^5^. The concept of Psychological Medicine, which has roots in the history of medicine itself, was included in the first edition of “Diagnostic and Statically Manual, Mental Disorder” (DSM-1) in 1952 under the heading “Psychosomatic Disorders” and in DSM-II in 1968 under the heading “Psycho Physiological Autonomic and Visceral Disorder.” In the 1980s, DSM-III replaced the phrase “Psychological Factor Affecting the Physical Conditions”^6^.

The DSM-II (1968) describes Psychosomatic Disorders as psychosomatic symptoms that affect a single organ system and are typically innervated by autonomic nerves^6^. Stress is a harmful or dangerous physiological response that is triggered by negative external factors (stressors)^7^. The most frequent oral lesions and signs of head-and-neck psychosomatic disorders associated with stress include Aphthous ulcers, Oral Lichen Planus (OLP), Tempero-Mandibular Disorders (TMD'S), Burning Mouth Syndrome (BMS), Atypical Facial Pain, Myofacial Pain Dysfunction Syndrome (MPDS), Dysgeusia, Glossopyrosis, Attrition, Xerostomia, and Halitos. Numerous investigations have demonstrated the complex nature of the etiological variables producing various illnesses^8^ Salivary Alpha-Amylase (sAA) sensitivity to changes in blood catecholamine values has only been indirectly demonstrated in a small number of studies, acting as a proxy marker of Sympatho-Adrenal Medullary System (SAM) activity reflecting the alterations during acute psychosocial stress^9^-^11^. sAA the enzyme found in saliva, is primarily responsible for the oral cavity's starch digestion. Only in the last ten years has this enzyme come under greater scrutiny as a Sympathetic Nervous System (SNS) stress marker. It has been demonstrated that sAA rises in response to acute stress^12^ and is associated with an increase in norepinephrine levels in the blood^13^. Women are also more susceptible to mental health issues as a result of the hormonal changes that coincide with the menopause. Stress can cause or exacerbate conditions like depression and sleeplessness because of the intimate relationship between the reproductive and stress axes^14^. Post-menopause decline in estrogen levels affects both the neurotransmitters serotonin and norepinephrine which are thought to be most directly associated with depression^15^.

sAA was identified as a promising option in the hunt for a readily available, non-invasive measure of SAM activation because of its strong neuro-hormonal regulation of secretion. sAA measurements have a key benefit over other metrics that indicate the activity of the SAM system because they are assessed in saliva, which enables ecological immediately apparent assessments^16^. It is recommended to use salivary biomarkers as a reliable, non-intrusive, and factual method for measuring acute psychosocial stress. They also aid in determining how important a role stress plays in the development or escalation of a wide range of health issues. sAA is one such biomarker that may be used to examine the human body's physiological response to stress especially when is of short duration and acute in nature^9^,^17^-^19^. The current study attempts to assess post-menopausal women's SAA levels as biochemical markers of acute stress

## Methods

The sample size consisted of 200 women subjects of more than 45 years, with a minimum of 2 years of post-menopausal history. 100 post-menopausal women without psychosomatic disorders; with no history of local or systemic diseases and who were not under any medication consisted of the control group and 100 post-menopausal women with psychosomatic disorders consisted of the study group as listed in Table 3. The case and the control groups were age-matched. Subjects under medication, those with a background of enduring psychiatric conditions and those currently receiving psychiatric treatment were not included in the research.

A comprehensive examination of the oral cavity and a thorough case history was recorded after the participants in the study provided their Informed Consent. An extensive intra-oral, as well as extra-oral examination, was performed to document the presence of a Psychosomatic condition in the Case Group. Both the Principal Investigator and the Co-investigator examined the study subjects to prevent bias. The study group included subjects with a favorable history of stress and those who met the criteria for disorders related to stress.

### Saliva Collection

The “Spit Technique” was used to obtain saliva from the patients without salivary stimulation. One hour preceding sample collection, the study subjects were observed not to drink, eat nor smoke. They were then positioned in the dental chair, theirheads forward, and instructed not to speak to avoid swallowing any saliva. Next, the participants were taught to periodically expel their saliva into a sterile measuring container for 8 to 10 minutes. The collected sample contained fluid from the entire oral cavity. To reduce diurnal variance, the collection of saliva was restricted to the timeframe of 9:00 am to 11:00 am. After collecting the sample, it was centrifuged for 10 minutes at 3000 rpm. The resulting supernatant was then collected and stored at -20°C. The analysis of sAA was performed using the ELISA method at the University Central Research Laboratory, using the ELISA Kit (LDN Labor Diagnostika Nord GmbH & Co. KG) following the procedure mentioned in the datasheet received with the kit.

### Principle

The procedure was performed according to the protocol sheet received with the ELISA Kit (LDN Labor Diagnostika Nord GmbH & Co. KG). Here 10 microliter of saliva was used as a sample. 10 microliter of saliva was used as the sample which was directly used after centrifugation. The procedure was performed using the microplate received with the kit. The short-chain p-nitrophenyl oligosaccharide and 2-chloro-4-nitrophenyl-alpha-maltotrioside (CNP-G3) of glucose polymers are hydrolyzed by the human alpha-amylase to form 2-chloro-4-nitrophenol (CNP). The rise in retardation is spectrophotometrically determined at 405 nm using LISA Plus microplate reader and is associated with the quantity of sAA activity present in the sample. 10 microliters of processed Saliva was mixed with 300 microliter of the reagent-A provided with the kit and immediately incubated at 37°C for 3 minutes. After the incubation is complete, the microplate is placed on a microplate reader at room temperature (22°–28° C) and the absorbance variation (A) is read twice at 405 nm, the first after one minute and the second after five minutes from the end of incubation time, subtracting each time the absorbance of the blank. Each sample was analysed with replicates to ensure the consistency of the result.

### Statistical analysis

The acquired data was entered into a Microsoft Excel spreadsheet. and analyzed using IBM SPSS Statistics, Version 22(Armonk, NY: IBM Corp). Mean and standard deviation were used for depicting descriptive data. An independent sample t-test was used to compare the duration and age of the research groups. Pearson's correlation test was employed to examine the relationship between sAA levels and tobacco consumption. Statistical significance was defined as a P value of < 0.05.

## Results

**Graph 1:**
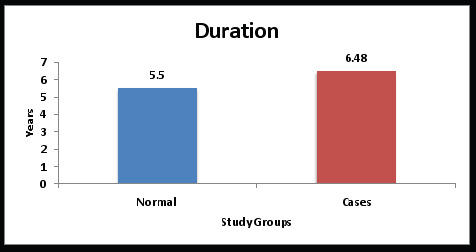
Comparison of post-menopause duration between the study groups

The study groups were compared based on age and duration using an independent sample t-test. The subjects in the control group ranged in age from 46 to 69 years old, with an average age of 56.61 years. The patients in the study ranged in age from 46 to 70, with an average age of 57.79 years. (Table 1 and Graph 1). However, the difference in age between the two groups was not statistically significant (Table 1). Pearson's correlation test was utilized for correlation between the study parameters in each study group. Statistical significance was defined as a P value of 0.05 (Table 1, Scatter chart 1) The findings were statistically significant since they demonstrated a rise in sAA levels with longer post-menopausal duration (Table 1, Graph 1). To depict the distribution, the levels of sAA have been shown on a scattered chart (Scatter chart 1).

**Table 1:** Correlation between the study parameters in each study group

Group			Age	α-amylase
**Normal**	**Duration**	**r**	0.56	0.15
		**p-value**	<0.001*	0.14(NS)
	**sAA(U/ml)**	**r**	-0.16	1
		**p-value**	0.10(NS)	
**Cases**	**Duration**	**r**	0.60	0.16
		**p-value**	<0.001*	0.11(NS)
	**sAA(U/ml)**	**r**	0.04	1
		**p-value**	0.67(NS)	

Pearson's correlation test

p<0.05 statistically significant

p>0.05 non-significant, NS

**Figure:**
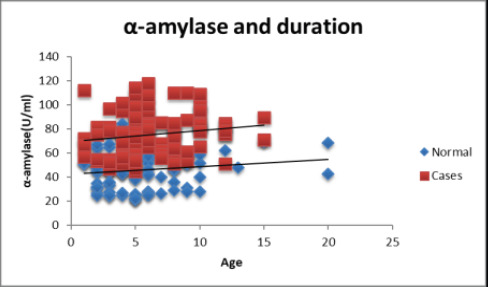
Scatter Chart 1

**Graph 2:**
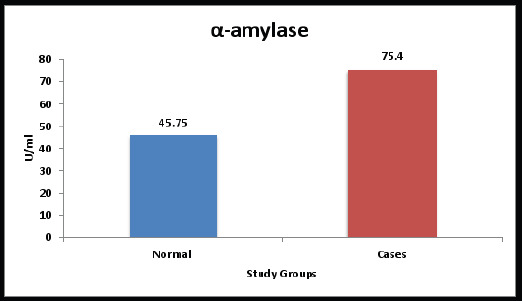
Comparison of sAA between the study groups

The mean salivary sAA levels in the control group and study group were 45.75 U/ml and 75.40 U/ml, respectively. The comparison between the groups showed that mean values were statistically highly significant. (Table 2, Graph 2)

**Table 2:** Comparison of sAA between the study groups

	Group	N	Mean	SD	Mean difference (95% CI)	t	df	p-value
**α-amylase**	**Normal**	100	45.75	14.91	-29.64 (-34.21, -25.07)	-12.78	198	<0.001*
	**Cases**	100	75.40	17.76				

Independent sample t-test p<0.05 statistically significant, p>0.05 non-significant, NS

The relationship between sAA levels and the psychosomatic disorders of the head and neck was investigated. For each disorder that met the inclusion criteria, mean values and standard deviations were obtained (Table 3). Mean sAA levels were seen to be highest among halitosis subjects (83.90 U/ml) and lowest among Burning mouth syndrome [BMS]/Glossopyrosis subjects (56.91 U/ml).

**Table 3:** Mean values of sAA for various Psychosomatic Disorders

Condition		sAA (U/ml)	
	N	Mean	SD
Halitosis	1	**83.90**	.
MPDS	14	82.17	16.83
TMD's	15	80.60	19.19
Atypical facial pain	13	71.81	12.31
Burning mouth syndrome [BMS]/Glossopyrosis	3	71.43	14.52
Aphthous stomatitis	4	63.98	12.87
OLP	8	63.96	11.18
Attrition	1	63.17	.
Dysgeusia	2	61.17	3.15
Xerostomia	1	**56.91**	.
Multiple	38	77.33	20.02
Total	100	75.40	17.76

## Discussion

One of the most thoroughly investigated topics of interest by psychophysiologists is the influence of psychosocial elements, especially stress, in the alterations of the human body. Stress and the factors that cause it are of a multidimensional nature^20^. For accurate depiction, multiple stress indicators throughout multiple stress-related mechanisms should be reviewed. sAA enzyme suggestive of SNS function has drawn more attention in recent years as a stress marker hence making it an important and accurate stress indicator of Hypothalamic-Pituitary-Adrenal (HPA) axis function.

In light of the aforementioned investigations, links between sAA and stress have been repeatedly discovered and attempted to be proven. Studies that demonstrated a relationship between anxiety and higher values of sAA have also been done, as noted in studies by Rohleder N et al.^11^, Nater et al.^12^, and Vineetha et al.^21^. However, no research on postmenopausal women with psychosomatic diseases has been conducted to examine sAA levels as stress markers. In this study, we aimed to use sAA as a stress marker to demonstrate a relationship between stress and psychosomatic head and neck disorders in postmenopausal women.

One of the areas that psycho-physiologists are particularly interested in researching is the part of stress as a psychosocial variable causing the changes in the human body. Nater UM et al.^19^ conducted studies that revealed sAA activation caused by psychological stress is a sign of sympathetic activity. This study intended to establish the efficacy of the enzyme sAA as a stress biomarker in postmenopausal women. Stress often causes significant harm to an individual's physical and mental wellness and may result in a number of illnesses. As previously indicated, numerous serum and salivary studies have established the function of sAA in acute stress.

There have been many other studies conducted by researchers^22^-^24^, where verbal as well as self-reported assessments were employed alone or in conjunction to assess stress. Further research is required as a result of the studies' highly contradictory findings, which are probably caused by the patient's clear mental and behavioral changes. People who feel stress and the problems that go along with it frequently have a tendency to either ignore or inflate their actual situation, influencing the study and distorting the results. This could be one of the key causes for the inconsistent findings of many earlier studies looking at the influence of psychosocial dynamics on the symptoms of head and neck disorders^21^.

In the current investigation, the mean sAA values in the control and study groups were 45.75 U/ml and 75.40 U/ml respectively. The study group had a higher acute stress factor, as evidenced by the findings of the comparison of the mean values across the groups, which were greatly statistically significant. Vineetha M et al.^21^ study is one of many that have sought to correlate stress and oral lesions in the past, However, few studies have demonstrated the occurrence of a variety of psychosomatic diseases affecting the head and neck in stressed women post-menopause. We have attempted to relate sAA to post-menopausal women's acute stress markers in this study.

This study explored the association between sAA and other Psychosomatic Disorders while providing mean and standard deviation for every psychosomatic disorder that affects the head and neck that were included in the inclusion criteria. (Table 3). Due to the interrelated etiology and positive feedback cycle between stress and psychosomatic diseases, 38% of the participants had numerous clinical manifestations of psychosomatic disorders.

Walsh N. et al.^25^, and Skosnik PD et al. [26] and Bosch JA et al.^27^,^28^ conducted investigations that were comparable to our study and found that sAA levels were elevated in response to physical stress or exercise and psychological stress. In a study by Bosch JA et al.^29^, it was found that the Autonomic Nervous System was more affected by stresses when it was activated, which is consistent with increased sAA levels in our study group. However, none of the aforementioned research examined any possible links between sAA and head and neck psychosomatic diseases which is an additional value of our study.

An objective assessment of psychological distress aids in determining the crucial role that stress plays in the emergence of a wide range of health issues as well as the appropriate course of action^30^. In our research, the mean value sAA levels were found to be lowest in participants with glossopyrosis (56.91 U/ml) and greatest in those with halitosis (83.90 U/ml) which is associated with acute stress. Although it is disputed to what extent sAA signals parasympathetic nervous system activity, it is frequently utilised in scientific research on acute stress to evaluate sympathetic nervous system activation^31^. This study, which is a rarity and the first of its kind, links sAA to acute stress as well as numerous Oral Psychosomatic problems that plague post-menopausal women. However, it is essential to keep in mind that sampling techniques can impact the content of saliva and, as a consequence, the levels of some of the most significant stress-linked analytical substances under study^32^.

The present study did not analyse sAA based on diurnal variations. Also, the sample size can be increased for each psychosomatic disorder and research can be focussed on them individually as seen in Table 3 which intends to contribute as an overview of the research findings which may serve as baseline data for further research. Future investigation in this area has a lot of room to examine the different forms, contributors, and origins of stress that result in psychosomatic diseases. A study in which beta-adrenergic antagonists suppressed an increase in sAA activity in response to a cold-water stressor^33^ provided the first proof that acute stress induces alteration in sAA activity. It is recommended that dental practitioners properly analyse and guide each patient both before and after any treatment, especially in postmenopausal/geriatric women.

## Conclusion

Stress is an inevitable part of lifespan. Whenever people ask for assistance, they are frequently coping with problems, stresses, and other aspects of their lives that make them feel exhausted both mentally and physically. Many people, particularly senior patients, believe they lack the tools or abilities needed to cope with the substantial amounts of pressure they are currently facing. The findings of this study suggest that post-menopausal women undergo routine stress screening. Salivary Alpha-Amylase analysis will help to diagnose those people who do not disclose their psychological suffering. Additionally, so that the patient may receive care and alleviation, stomatologists must thoroughly evaluate the head and neck for any complications associated with stress.
